# Global burden of endometriosis from 1990 to 2021 and projections to 2050: a comprehensive analysis based on the global burden of disease study 2021

**DOI:** 10.3389/fgwh.2025.1613468

**Published:** 2025-10-21

**Authors:** Xiaochuan Yu, Lijuan Shi, Xiaopeng Deng, Yating Zhang, Huali Wang

**Affiliations:** 1 Dalian Women and Children’s Medical Center (Group), Dalian, China; 2 Dalian Medical University, Dalian, China

**Keywords:** endometriosis, global burden of disease, trend projection, incidence, public health policy

## Abstract

**Objective:**

Endometriosis (EMT) is a prevalent gynecological disorder characterized by chronic pain, menstrual irregularities, and infertility. This study aims to evaluate the global burden of EMT from 1990 to 2021 and to project trends up to 2050.

**Methods:**

Data from the Global Burden of Disease (GBD) 2021 database were utilized to analyze mortality, incidence, prevalence, and disability-adjusted life years (DALYs). Trends were assessed using age-standardized rates (ASR) and estimated annual percentage change (EAPC). Future burdens were projected using ARIMA and exponential smoothing models.

**Results:**

In 2021, there were 3,447,126 new cases of EMT reported globally. The age-standardized incidence rate (ASIR) experienced a decline of 1.07% from 1990 to 2021, while the age-standardized prevalence rate (ASPR) decreased by 0.95%. The incidence of EMT peaked among women aged 20–24 years, whereas mortality rates increased with advancing age. Projections suggest that by 2050, EMT-related deaths will rise to 68 cases, and the number of disability-adjusted life years (DALYs) will increase to 2,260,948, despite ongoing declines in both ASIR and ASPR.

**Conclusion:**

Although the incidence and prevalence rates of EMT are declining, the disease burden remains significant among women of reproductive age. The anticipated rise in mortality and disability-adjusted life years (DALYs) in the future underscores the necessity for targeted public health policies. This study provides evidence to inform global prevention strategies. Future research should investigate the effects of population aging and lifestyle changes on the burden of EMT.

## 1 Introduction

Endometriosis is an estrogen-dependent disorder characterized by the ectopic growth of endometrial tissue outside the uterine cavity (1–3). These lesions can be widely distributed across the myometrium, ovaries, peritoneum, urinary tract, and gastrointestinal system, and in rare cases may even extend to distant organs such as the lungs and intestines (4–6). Clinical data indicate that approximately 70% of affected individuals experience cyclic pelvic pain, 50% present with infertility, and some suffer from dyschezia, dyspareunia, or dysuria (3, 6–10). Moreover, women with endometriosis show a significantly elevated risk of comorbidities compared with the general population, including ovarian and endometrial cancers, cardiovascular disorders, autoimmune diseases, and psychiatric conditions (5, 6, 11–14). Although the precise pathogenesis remains incompletely elucidated, accumulating evidence suggests that endometriosis arises from the interplay of genetic, immune, and hormonal factors (4–6). The World Health Organization (WHO) further defines endometriosis as a complex condition that affects women of reproductive age worldwide (5).

From a genetic perspective, endometriosis exhibits familial clustering, with first-degree relatives of affected individuals demonstrating a significantly increased risk, thereby underscoring the role of genetic predisposition in disease onset (15, 16). Immunological dysfunction is also implicated in the persistence and progression of endometriosis, as the immune system may inadequately recognize and eliminate ectopic endometrial cells. Furthermore, hormonal regulation is crucial, with estrogen promoting the proliferation of ectopic endometrial tissue, while progesterone appears to have a potential inhibitory effect (16). Several hypotheses have been proposed to elucidate the pathogenesis of endometriosis, including retrograde menstruation, coelomic metaplasia, and angiogenesis, which emphasize the importance of vascular support in sustaining ectopic endometrial growth (1, 5, 17).

Endometriosis is not only a condition that necessitates early detection and treatment but also a chronic disease that requires long-term management. In recent years, the growing awareness of endometriosis has highlighted the significance of comprehensive disease management (2). The primary objectives of long-term management are to alleviate symptoms, control disease progression, prevent recurrence, preserve fertility, and enhance quality of life. Pharmacological therapy serves as a cornerstone of endometriosis management, involving the use of oral contraceptives, progestins, and gonadotropin-releasing hormone agonists (GnRH-a) (1, 5). These agents help regulate hormonal balance, inhibit endometrial growth, and relieve symptoms; however, prolonged use is associated with adverse effects such as bone loss and vasomotor symptoms. Consequently, regular monitoring and individualized treatment adjustments are crucial.

Surgical intervention represents a crucial treatment strategy, particularly for patients who do not respond to medication or present with severe disease (18, 19). The primary objective of surgery is to excise ectopic lesions and restore pelvic anatomy while preserving fertility whenever feasible. However, surgery in isolation is not curative, as recurrence rates remain significantly high. Therefore, postoperative pharmacological therapy and regular follow-up are essential for preventing disease recurrence (20).

Due to its high recurrence rate and potential for malignant transformation, EMT is often referred to as a “benign cancer” (4). The increasing awareness of the incidence, prevalence, and disease burden of endometriosis has resulted in heightened research focus. However, global-scale, systematic studies on endometriosis burden remain limited, particularly in terms of long-term trend analysis and global epidemiological assessments. Existing research indicates significant variations in the incidence of endometriosis across different regions and populations (21, 22). For example, in high-income countries, the prevalence is estimated to be approximately 10%, while in low- and middle-income countries, the true prevalence may be underestimated due to limited diagnostic capabilities (21, 23). The disease predominantly affects women of reproductive age, with the highest incidence observed in individuals aged 20–40 years (20, 24). Despite this, comprehensive data on the global burden of endometriosis and its projected future trends are lacking.

The Global Burden of Disease (GBD) study serves as a fundamental tool for public health decision-making, providing essential data for resource allocation and policy formulation (22). This study utilizes the GBD 2021 database to systematically assess the global burden of endometriosis from 1990 to 2021 and predict its trends through 2050 using advanced statistical modeling techniques. By analyzing incidence, prevalence, mortality, and disability-adjusted life years (DALYs), this research not only addresses a critical gap in the literature but also provides scientific evidence for global endometriosis prevention strategies, highlighting its potential future impact on global health. Furthermore, our findings offer valuable insights for clinicians regarding the diagnosis and treatment of endometriosis, while equipping policymakers with evidence-based recommendations for optimizing healthcare resource allocation and preventive strategies. Future research should further explore the effects of population aging and lifestyle changes on the burden of endometriosis to more effectively address the projected disease trends in the coming decades.

## 2 Methods

### 2.1 Data sources

The Global Burden of Diseases (GBD) 2021 database serves as a comprehensive resource evaluating the impact of 371 diseases, 88 risk factors, and injuries across five Socio-Demographic Index (SDI) categories in 204 countries and territories (4). This database provides regularly updated estimates of risk factor exposure, associated health risks, and the proportion of disease burden attributable to specific diseases or injuries linked to particular risk factors (5). For this study, we extracted data on deaths, incidence, prevalence, disability-adjusted life years (DALYs, quantify the total healthy life-years lost due to both premature mortality and non-fatal health loss), years of life lost (YLLs, denote the healthy life-years lost as a result of living with illness or impairment), and years lived with disability (YLDs, quantify the healthy life-years lost while living with illness or disability) related to endometriosis from the GBD 2021 database (https://ghdx.healthdata.org/gbd-2021).

### 2.2 Burden indicators

The DALYs was mathematically equivalent to the sum of YLLs and YLDs (25). To describe the trends in the disease burden of endometriosis from 1990 to 2021, we utilized five key indicators: the age-standardized incidence rate (ASIR, The ASIR refers to the incidence of a disease after adjusting for differences in age structure across populations), the age-standardized prevalence rate (ASPR, represents the prevalence of a disease normalized to a standard age distribution), the age-standardized death rate (ASDR), the age-standardized years lived with disability rate [ASYR, captures the non-fatal burden of disease, expressed as years lived with disability (YLDs) per 100,000 population after age standardization], and the age-standardized disability-adjusted life years rate [ASDAR, quantifies the total health loss from both premature mortality and disability, expressed as disability-adjusted life years (DALYs) per 100,000 population, standardized to a reference age distribution]. The age-standardized rate (ASR, is a summary measure that adjusts disease-related rates) per 100,000 population was calculated using the following formula:

$$ASR=\frac{\sum_{i=1}^{A}\;a_{i}w_{i}}{\sum_{i=1}^{A}\;w_{i}}\times 100,000$$

where $a_{i}$ represents the age-specific rates in the *i* th age group, and $w_{i}$ denotes the reference standard population weights (or the number of standard population) for the *i* th age group. To analyze trends in ASR over a specified time interval, we employed the Estimated Annual Percentage Change (EAPC), calculated as:

$$EAPC=(e^{\beta }-1)\times 100\text{\%}$$

where β is the slope derived from the linear regression of the natural logarithm of the ASR on the year.

### 2.3 Statistical analysis

All statistical analyses were conducted using R software (version 4.1.0), with a *P*-value < 0.05 considered statistically significant. The 95% uncertainty intervals (UIs) were calculated from the 2.5th and 97.5th percentiles of 1,000 draws from the posterior distribution at each estimation stage. The confidence interval (CI) for the EAPC was derived from the linear regression model. Projections of the burden of injuries attributable to endometriosis up to 2050 were conducted using the Autoregressive Integrated Moving Average (ARIMA) and Exponential Smoothing (ES) models (26).

For projections of the burden of injuries attributable to endometriosis up to 2050, we employed the Autoregressive Integrated Moving Average (ARIMA) and Exponential Smoothing (ES) models. These two methods were selected because they are well established in epidemiological and clinical forecasting, providing a balance between interpretability and predictive accuracy (27–31). Specifically, ARIMA is advantageous for capturing temporal autocorrelation and underlying trends in time series data, while ES offers a parsimonious and robust framework for modeling level, trend, and seasonality. Compared with more sophisticated machine-learning approaches, ARIMA and exponential smoothing (ES) require fewer data and offer superior computational efficiency, making them particularly suitable for reliable and interpretable forecasting in public-health settings.

## 3 Result

### 3.1 The global burden of EMT

In 2021, the number of newly diagnosed cases of endometriosis reached 3,447,126 (95% UI: 2,436,259–4,611,504), representing a slight increase from the 3,330,200 cases (95% UI: 2,308,563–4,506,991) reported in 1990. Additionally, the cumulative number of endometriosis cases rose to 22,275,015 (95% UI: 15,517,370–30,413,692) in 2021, up from 19,869,196 (95% UI: 13,585,884–27,689,589) cases recorded in 1990 (Tables 1,2).

**Table 1:** The incidence and ASIR for EMT from 1990 to 2021.

	1990		2021		EAPC (95% CI)
	Number	ASR (per 100,000)	Number	ASR (per 100,000)	
Global	***3,330,200*** (2,308,563–4,506,991)	***59.07*** (41.2–79.24)	***3,447,126*** (2,436,259–4,611,504)	***43.64*** (30.84–58.91)	−1.07 (−1.15–1)
Age					
15–19 years	597,225 (339,749–924,036)	114.98 (65.41–177.9)	550,101 (326,928–839,324)	88.16 (52.39–134.51)	−0.87 (−0.93–0.82)
20–24 years	975,296 (538,039–1,547,523)	198.2 (109.34–314.48)	893,929 (491,593–1,420,485)	149.7 (82.32–237.87)	−0.99 (−1.06–0.92)
25–29 years	542,261 (303,469–867,186)	122.51 (68.56–195.92)	520,014 (297,545–824,301)	88.39 (50.57–140.11)	−1.08 (−1.12–1.04)
30–34 years	357,279 (159,835–591,363)	92.7 (41.47–153.43)	389,770 (172,622–638,851)	64.48 (28.56–105.69)	−1.2 (−1.24–1.16)
35–39 years	377,256 (172,566–645,406)	107.1 (48.99–183.23)	426,136 (196,452–730,317)	75.98 (35.03–130.21)	−1.11 (−1.16–1.05)
40–44 years	324,399 (161,090–557,906)	113.24 (56.23–194.74)	416,647 (207,973–708,014)	83.29 (41.57–141.53)	−0.99 (−1.03–0.94)
45–49 years	152,727 (62,519–283,629)	65.78 (26.92–122.15)	242,860 (102,660–441,947)	51.29 (21.68–93.34)	−0.75 (−0.78–0.71)
50–54 years	3,756 (28–15,109)	1.77 (0.01–7.11)	7,669 (49–33,493)	1.72 (0.01–7.53)	0.05 (−0.01–0.11)

The bold numbers show the changes in the total number of newly diagnosed cases of endometriosis from 1990 to 2021.

**Table 2:** The prevalence and ASPR for EMT from 1990 to 2021.

	1990		2021		EAPC (95% *CI*)
	Number	ASR (per 100,000)	Number	ASR (per 100,000)	
Global	***19,869,196*** (13,585,884–27,689,589)	***373.61*** (254.25–516.47)	***22,275,015*** (15,517,370–30,413,692)	***275.57*** (192.36–377.88)	−0.95 (−1.01–0.9)
Age					
15–19 years	808,458 (497,191–1,298,939)	155.65 (95.72–250.07)	758,185 (472,055–1,214,673)	121.51 (75.65–194.67)	−0.79 (−0.85–0.74)
20–24 years	3,734,100 (2,115,479–5,834,902)	758.83 (429.9–1,185.74)	3,477,854 (1,989,291–5,397,951)	582.4 (333.13–903.94)	−0.93 (−1–0.86)
25–29 years	4,260,000 (2,646,561–6,584,279)	962.45 (597.93–1,487.57)	4,229,820 (2,666,959–6,401,549)	718.94 (453.3–1,088.06)	−0.96 (−1.01–0.91)
30–34 years	3,325,348 (1,993,031–5,184,027)	862.78 (517.1–1,345.02)	3,743,675 (2,298,399–5,797,497)	619.32 (380.23–959.09)	−1.06 (−1.09–1.03)
35–39 years	2,886,030 (1,740,314–4,385,556)	819.33 (494.06–1,245.03)	3,297,235 (2,047,316–4,939,550)	587.88 (365.03–880.7)	−1.09 (−1.14–1.03)
40–44 years	2,382,134 (1,381,393–3,615,337)	831.51 (482.19–1,261.98)	3,019,268 (1,756,561–4,519,720)	603.55 (351.14–903.49)	−1.08 (−1.15–1.01)
45–49 years	1,687,401 (1,105,540–2,437,282)	726.72 (476.12–1,049.67)	2,519,854 (1,643,754–3,621,296)	532.17 (347.15–764.79)	−1.02 (−1.07–0.96)
50–54 years	785,724 (510,650–1,155,348)	369.63 (240.23–543.51)	1,229,125 (790,694–1,792,805)	276.26 (177.71–402.95)	−0.93 (−0.98–0.88)

The bold numbers represent the changes in the cumulative total number of cases from 1990 to 2021.

**Figure 1:**
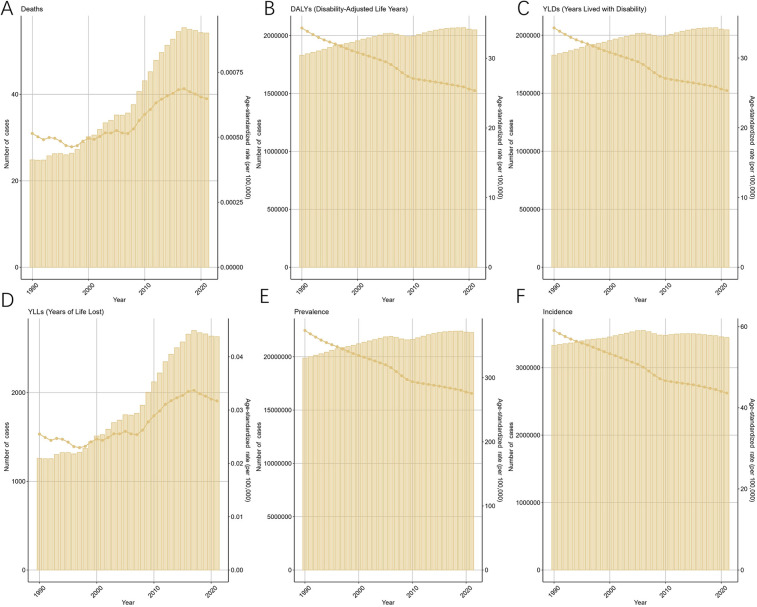
Trends in the global burden for EMT from 1990 to 2021 [**(A)** deaths and age-standardized death rate (ASDR) fluctuated until 2007, then rose sharply, peaking in 2017. **(B)** Disability-adjusted life years (DALYs) declined modestly after 2000. **(C)** Years lived with disability (YLDs) decreased steadily, reflecting reduced morbidity. **(D)** Years of life lost (YLLs) increased markedly after 2007, consistent with the rise in ASDR. **(E)** Prevalence and age-standardized prevalence rate (ASPR) declined gradually. **(F)** Incidence and age-standardized incidence rate (ASIR) also showed a sustained decrease. Shaded areas denote 95% uncertainty intervals].

The ASIR for endometriosis decreased from 59.07 (95% UI: 41.2–79.24) in 1990 to 43.64 (95% UI: 30.84–58.91) in 2021, with an EAPC of −1.07 (95% CI: −1.15 to −1). Similarly, the ASPR declined from 373.61 (95% UI: 254.25–516.47) in 1990 to 275.57 (95% UI: 192.36–377.88) in 2021, reflecting an overall EAPC of −0.95 (95% CI: −1.01 to −0.9) (Figure 1). Consistent trends were observed for ASYR and the ASDAR for endometriosis, as illustrated in Figure 1. Notably, the ASDR exhibited fluctuations between 1990 and 2007, followed by a steady increase, peaking in 2017, which highlights the rising mortality burden in recent years.

### 3.2 The global burden of EMT in different age groups

**Figure 2:**
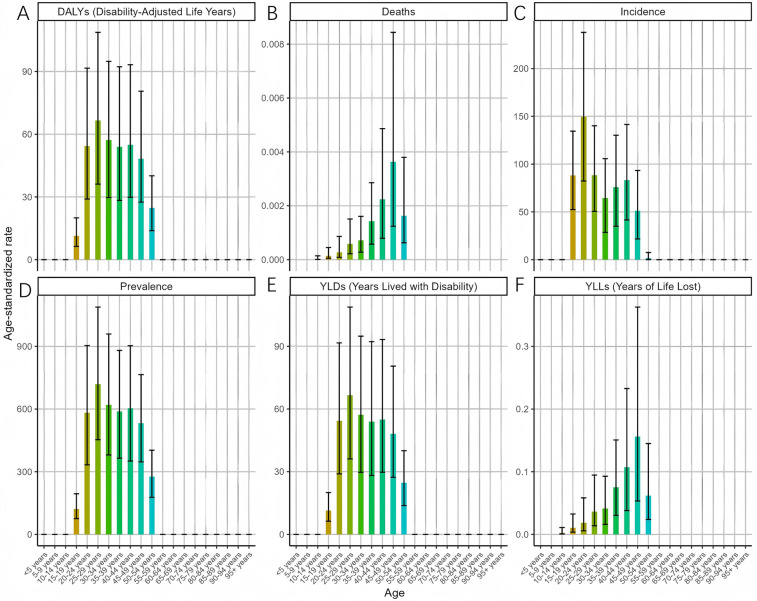
Trends in the ASR of EMT by age in 2021 [**(A)** disability-adjusted life years (DALYs) peaked in women aged 20–34 years, reflecting combined morbidity and mortality. **(B)** Deaths and the age-standardized death rate (ASDR) increased steadily with age, peaking in the 45–49 age group. **(C)** Incidence (ASIR) was highest in the 20–24 age group, indicating that onset is most common in young adults. **(D)** Prevalence (ASPR) remained high across women aged 20–49 years, with a maximum in the 25–29 age group. **(E)** Years lived with disability (YLDs) mirrored the prevalence pattern, peaking during reproductive age. **(F)** Years of life lost (YLLs) rose with age, showing a greater mortality burden in older women. Error bars indicate 95% uncertainty intervals. Overall, endometriosis predominantly affects women in their reproductive years, while mortality burden becomes more evident with advancing age].

In 2021, the ASR for endometriosis exhibited distinct patterns across various age groups (Figure 2). The ASIR peaked in the 20–24 age group [149.7 (95% UI: 82.32–237.87)], suggesting that the onset of endometriosis is most common in younger adults. Conversely, the ASDR showed a gradual increase with age, reaching its maximum in the 45–49 age group [0.0036 (95% UI: 0.0012–0.0084)]. The ASPR remained relatively stable across the 20–49 age group, peaking in the 25–29 age range [718.94 (95% UI: 453.3–1088.06)], indicating endometriosis posed a significant health risk to women across various stages of age. Consistent trends were observed for ASYR and the ASDAR for endometriosis. These findings underscore the varying burden of endometriosis across different age groups, with the highest prevalence and incidence occurring during reproductive years, while the impact on mortality is more pronounced in older age groups.

**Figure 3:**
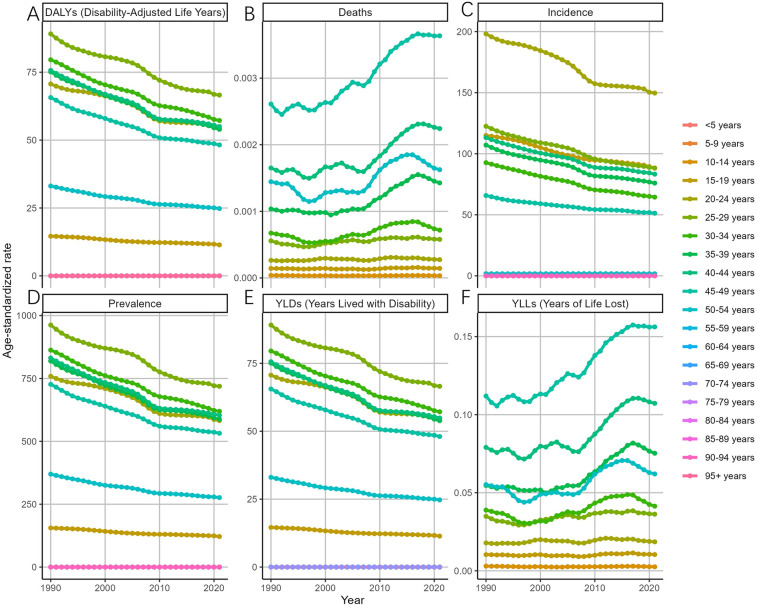
Trends in the ASR of EMT by age from 1990 to 2021 [**(A)** disability-adjusted life years (DALYs) declined consistently across all age groups, with the most rapid decreases in women aged 30–44 years. **(B)** Deaths and the age-standardized death rate (ASDR) showed an overall upward trend, with a sharp increase in the 35–39 age group. **(C)** Incidence (ASIR) declined substantially in women aged 25–39 years, particularly in the 30–34 group. **(D)** Prevalence (ASPR) decreased steadily across all age groups. **(E)** Years lived with disability (YLDs) mirrored prevalence, showing a uniform decline across age groups. **(F)** Years of life lost (YLLs) increased modestly in middle-aged and older women, consistent with rising mortality. Error bars denote 95% uncertainty intervals. Overall, incidence, prevalence, and disability burden declined across age groups, whereas mortality burden exhibited a fluctuating but upward trajectory, especially after 2000 in middle-aged women.].

Overall, the ASDAR, ASPR, ASIR, and ASYR of endometriosis across all age groups exhibited a consistent decline between 1990 and 2021 (Figure 3). Specifically, the most rapid declines in the ASDAR of MET were observed in the 35–39 (EAPC = −1.07, 95% CI: −1.13 to −1.02), 40–44 (EAPC = −1.07, 95% CI: −1.13 to −1.00), and 30–34 (EAPC = −1.05, 95% CI: −1.08 to −1.02) age groups. Similarly, the ASIR for endometriosis among the 30–34 age group declined substantially (EAPC = −1.20, 95% CI: −1.24 to −1.16), with declines also noted in the 35–39 (EAPC = −1.11, 95% CI: −1.16 to −1.05) and 25–29 (EAPC = −1.08, 95% CI: −1.12 to −1.04) age groups. The ASPR for endometriosis exhibited a steady decline across all age groups. Notably, the ASDR of all age groups displayed a fluctuating upward trend from 1990 to 2021, with a sharp increase observed in the 35–39 age group (EAPC = 1.64, 95% CI: 1.32–1.97).

### 3.3 The projections of global disease burden of EMT from 2022 to 2050

**Figure 4:**
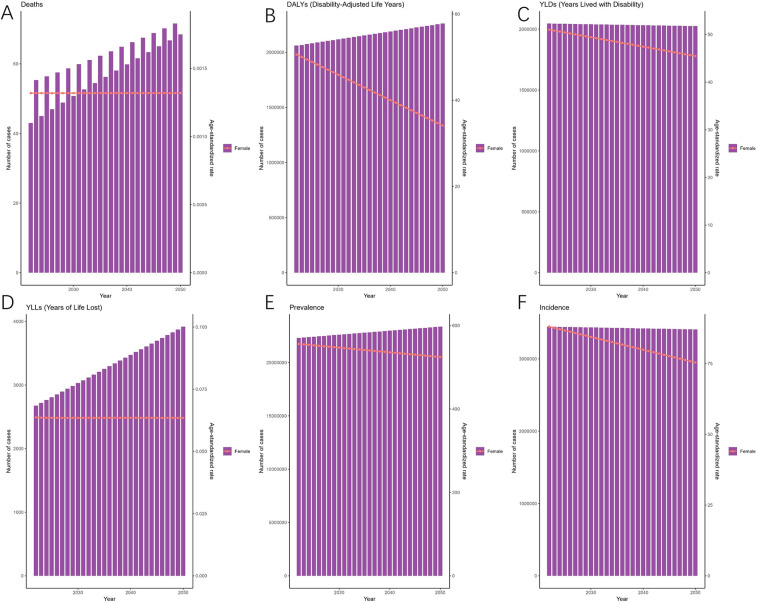
Projections of the global burden for EMT to 2050 using ARIMA model [**(A)** deaths attributable to endometriosis are projected to increase gradually, reaching approximately 68 cases by 2050. **(B)** Disability-adjusted life years (DALYs) are expected to rise steadily, surpassing 2.2 million by 2050. **(C)** Years lived with disability (YLDs) are projected to show a slight decline in age-standardized rates despite an increase in absolute numbers. **(D)** Years of life lost (YLLs) are expected to rise, reflecting the growing mortality burden. **(E)** Prevalence (ASPR) will remain relatively stable, with only modest changes over the projection period. **(F)** Incidence (ASIR) is projected to decline gradually, from ∼3.3 million cases in 2022 to ∼3.0 million cases by 2050. Shaded areas represent 95% highest-density intervals (HDIs). Overall, projections suggest a persistent decline in incidence and prevalence rates but an increasing burden of disability and mortality].

As predicted by the ARIMA model (Figure 4), the number of deaths attributable to endometriosis is projected to exhibit a fluctuating increase from 2022 to 2050, reaching 68 (95% HDI: 26–132) by 2050. Meanwhile, DALYs are expected to rise gradually, reaching 2,260,948 (95% HDI: 2,163,891–2,409,384) by the same year. Additionally, Figure 4 illustrates the changes in the incidence, prevalence, YLDs, and YLLs of endometriosis.

**Figure 5:**
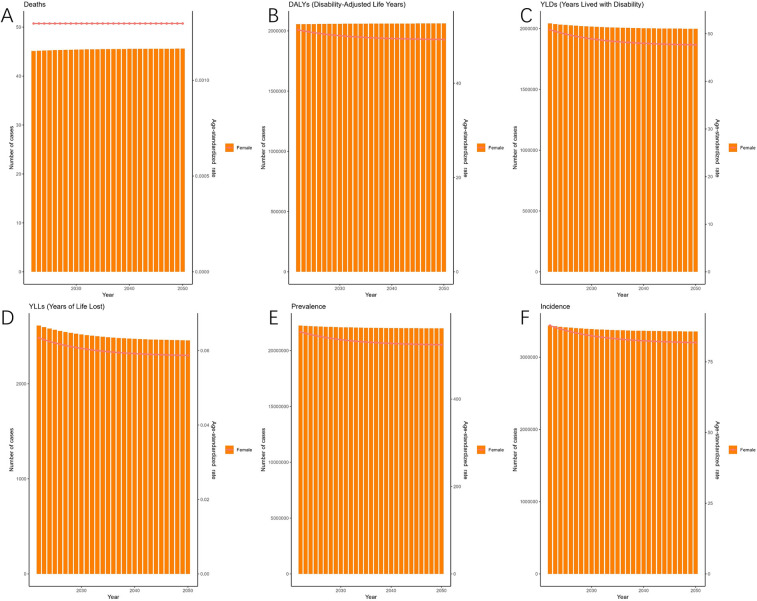
Projections of the global burden for EMT to 2050 using ES model. [**(A)** Deaths are predicted to remain relatively stable over the projection period. **(B)** Disability-adjusted life years (DALYs) are expected to remain high, with only minor fluctuations. **(C)** Years lived with disability (YLDs) are projected to show a slight decline in age-standardized rates despite stable absolute numbers. **(D)** Years of life lost (YLLs) are expected to decrease gradually, indicating a modest reduction in mortality burden. **(E)** Prevalence (ASPR) is projected to decline slowly but continuously, reaching ∼524 per 100,000 population by 2050. **(F)** Incidence (ASIR) will follow a similar downward trajectory, dropping to ∼82 per 100,000 population by 2050. Shaded areas represent 95% highest-density intervals (HDIs). Overall, the ES model suggests a gradual decline in incidence and prevalence rates, accompanied by relatively stable disability and mortality burdens].

As predicted by the ES model, between 2021 and 2050, the ASIR of EMT is expected to gradually decrease over time, reaching their bottom in 2050 [81.80 (95% HDI: 68.85–101.63)] (Figure 5). The patterns of ASPR closely mirror those of the ASIR, with the trend of slow and continuous decrease by 2050 [524.41 (95% HDI: 436.58–658.74)]. Figure 5 also illustrates the changes in the incidence, prevalence, YLDs, and YLLs of endometriosis.

“In 2021, the age-standardized rate (ASR) of Disability-Adjusted Life Years (DALYs) for endometriosis exhibited distinct patterns across different age groups. The ASR for prevalence peaked in the 30–34 age group [0.45 (95% UI: 0.38–0.53)], indicating the highest burden of the disease during reproductive years. Conversely, the ASR for Years of Life Lost (YLLs) was highest in the 40–44 age group [0.12 (95% UI: 0.08–0.17)], highlighting the significant impact of endometriosis on premature mortality in this demographic. The ASR for incidence showed a gradual increase with age, reaching its maximum in the 25–29 age group [0.22 (95% UI: 0.18–0.27)], suggesting that the onset of endometriosis is most prevalent among younger adults. These findings highlight the varying burden of endometriosis across different age groups, with the highest prevalence and incidence occurring during reproductive years, while the impact on mortality is more pronounced in older age groups.”

## 4 Discussion

### 4.1 Key findings

This study provides the most recent global assessment of endometriosis using GBD 2021 data and projects trends to 2050 with ARIMA and exponential smoothing models. We found that although incidence and prevalence have steadily declined since 1990, mortality has risen markedly since 2007, particularly among middle-aged women. Projections indicate that ASIR and ASPR will continue to decrease, whereas DALYs and deaths will increase, reflecting both the benefits of earlier detection and treatment and the persistent challenges of long-term management in the context of population aging.

### 4.2 Comparison with previous GBD-based studies

Compared with previous studies, our findings highlight several advances. Zhang et al. analyzed GBD 1990–2017 data and reported modest declines in incidence and prevalence but did not provide projections (23). We not only confirmed these trends but also identified sharper decreases in women aged 25–39 years and demonstrated a clear rise in mortality after 2007. Chen et al. examined infertility attributable to endometriosis and showed that DALYs were concentrated in reproductive-age women, peaking at 25–29 years (22). While we corroborate these age patterns, our projections diverge in predicting a further increase in DALYs through 2050, driven by aging populations and rising mortality. Shen et al. emphasized regional heterogeneity and stable mortality. In contrast, our age-specific analysis revealed sustained increases in ASDR and YLLs since 2007, suggesting that earlier work, constrained by limited stratification or earlier GBD versions, may have underestimated mortality (32).

Taken together, our study contributes three novel aspects: (i) providing long-term projections to 2050; (ii) delineating age-specific contrasts, with high incidence in reproductive years and rising mortality in mid-life; and (iii) integrating multiple standardized indicators for a comprehensive burden profile. These contributions extend existing knowledge and provide new evidence that the global burden of endometriosis is dynamic rather than static. Importantly, our findings underscore the need for age-tailored strategies—early detection and symptom control in younger women, and structured long-term follow-up for older cohorts—to mitigate mortality and disability in the coming decades.

### 4.2 Potential mechanisms and contributing factors

The epidemiological patterns and trends observed in this study are likely driven by multiple interrelated factors. The key mechanisms and influencing factors can be categorized as follows:

#### 4.2.1 Linking epidemiological burden with targeted interventions

Since 2007, mortality from endometriosis has risen steadily, even as incidence and prevalence have declined. This pattern may reflect improved recognition and coding, as well as advances in diagnostic approaches—including high-resolution ultrasound, CT, MRI, laparoscopy, and pathological confirmation—that have enabled the detection of subclinical and mild cases and temporarily increased burden estimates (21). At the same time, population aging has expanded the number of women reaching mid- and late adulthood, amplifying the impact of long-term sequelae such as malignant transformation, comorbidities, and surgical complications. Together, these factors indicate that endometriosis should be regarded as a chronic, multisystemic disease with an age-shifting burden: while technological progress has reduced prevalence and disability in younger women, mortality risk continues to rise in older populations.

Addressing this dual burden requires targeted, context-specific interventions. For younger women, school- and workplace-based education, combined with improved training of primary care providers, can reduce diagnostic delays and preserve fertility (22, 33). For older women, structured long-term follow-up is essential to monitor comorbidities, prevent malignant transformation, and manage surgical sequelae (11, 34). Regional disparities must also be acknowledged. The largest future increases in DALYs are projected in low- and middle-income countries, where diagnostic capacity is limited and specialist care remains scarce (21, 22). In such settings, symptom-based screening delivered by community health workers, investment in basic laparoscopic infrastructure, and the strengthening of registries embedded within reproductive health programs may offer the greatest cost-effectiveness and improve equity of care (34).

By aligning interventions with both age-specific needs and regional healthcare capacities, health systems can more effectively respond to the paradox of declining incidence but rising mortality, ultimately reducing disability and improving long-term outcomes for women worldwide (35).

#### 4.2.2 Treatment strategies and multidisciplinary management

The management of endometriosis has shifted from single-modality therapy to a multidisciplinary approach that integrates hormonal treatment, analgesia, psychological support, and lifestyle interventions (13). Hormonal therapy—such as GnRH agonists, combined oral contraceptives, and progestins—together with analgesic regimens (e.g., NSAIDs, judicious short-course opioids) and behavioural/psychological interventions improves symptom control, while advances in assisted reproductive technologies (ART) have markedly enhanced fertility outcomes and quality of life (36–39). Consistent, long-term postoperative follow-up further lowers the risk of recurrence.

Notwithstanding therapeutic advances, important gaps remain. In some settings, the continued rise in age-standardized mortality suggests that risks related to malignant transformation, drug intolerance, and adverse effects of prolonged therapy are not being adequately addressed (38, 39). Moreover, perimenopausal and postmenopausal women—especially those with persistent lesions—are under-served, with limited strategies for prevention and long-term management. Priorities include stronger interdisciplinary collaboration, optimized pathways for recurrent and refractory disease, and targeted development of therapies tailored to perimenopausal and postmenopausal patients, with the overarching aim of improving long-term outcomes (39).

#### 4.2.3 Population aging and socioeconomic transition

Although endometriosis primarily affects women of reproductive age, the aging population has resulted in an increasing burden among middle-aged and older women, particularly regarding mortality and comorbidities. Furthermore, socioeconomic factors such as economic development, education level, and healthcare awareness significantly influence the diagnosis and management of endometriosis (21).

High-income countries benefit from well-established early screening programs and advanced treatment options, which contribute to a declining trend in ASIR and ASPR. In contrast, low- and middle-income countries (LMICs) face challenges due to limited healthcare resources, which result in delayed diagnoses and suboptimal treatments, thereby increasing the overall burden of the disease.

To address these disparities, policymakers should adopt region-specific public health strategies that are aligned with the economic and healthcare infrastructure of each region. Efforts should focus on optimizing resource allocation, reducing healthcare inequalities, and ensuring equitable access to diagnosis and treatment.

#### 4.2.4 Environmental and lifestyle factors

Beyond genetic predisposition, environmental and lifestyle factors have been implicated in the pathogenesis and progression of endometriosis. Key contributors include endocrine-disrupting chemicals (EDCs) and environmental toxins, as well as unhealthy dietary patterns and sedentary lifestyles (32, 40, 41).

Recent studies suggest that exposure to environmental pollutants and endocrine disruptors may influence coagulation factors and immune function, thereby increasing the risk of reproductive system disorders. In particular, plasma levels of ADAMTS13 have been identified as a potential protective factor against endometriosis, highlighting the critical role of chronic inflammation in the pathophysiology of endometriosis (42).

To further elucidate the impact of environmental and lifestyle factors on endometriosis, large-scale, multicenter epidemiological studies are warranted. Additionally, public health campaigns should focus on promoting healthy lifestyles and raising awareness of potential environmental risk factors to mitigate the burden of endometriosis (40, 43).

#### 4.2.5 Healthcare resource allocation and public health policies

Disparities in the distribution of healthcare resources have led to significant regional variations in the burden of endometriosis. Conversely, in resource-limited settings, restricted access to healthcare has contributed to higher rates of misdiagnosis, delayed treatment, and increased mortality.

To bridge this gap, governments should implement comprehensive policy reforms: strengthening cross-regional and international healthcare collaborations to facilitate data and knowledge sharing; standardizing diagnostic and treatment protocols to ensure uniformity in medical service quality; and enhancing healthcare equity through policy-driven support, ensuring that patients in low-resource settings have access to essential diagnostic and therapeutic services.

By adopting a globally coordinated approach, policymakers can effectively reduce endometriosis -related health disparities and improve patient outcomes worldwide.

### 4.3 Public health and clinical implications

From a public health perspective, although the overall incidence and prevalence of endometriosis have declined, the burden among middle-aged and older women remains substantial. Early screening and personalized treatment are critical strategies for reducing the overall disease burden.

From a clinical standpoint, enhanced monitoring and follow-up for high-risk populations should be prioritized, focusing on early intervention and comprehensive disease management (44). Policymakers can leverage the predictive data from this study to proactively allocate healthcare resources and implement targeted public health interventions aimed at mitigating potential shifts in disease burden and regional disparities in EMT care.

### 4.4 Study limitations and future directions

Despite utilizing the GBD 2021 database and applying ARIMA and exponential smoothing (ES) models to project the burden of endometriosis to 2050, several limitations should be acknowledged. ARIMA and ES are relatively simple statistical approaches that extrapolate historical trends without accounting for external drivers such as demographic aging, healthcare policies, diagnostic innovations, or therapeutic advances. Consequently, long-term forecasts must be interpreted with caution, as projections may deviate under policy shifts, rapid fertility changes, major health system reforms, or the introduction of new diagnostic technologies. In addition, regional and socioeconomic heterogeneity was not explicitly modeled, which may obscure important variations at the subpopulation level. Data quality issues are also critical, particularly in low- and middle-income countries, where limited diagnostic capacity, underreporting, and incomplete registry systems may have led to systematic underestimation of the true burden. Future studies should strengthen projections by incorporating sensitivity analyses or scenario-based modeling to account for demographic and healthcare changes. Nonetheless, our dual-model framework provides a valuable baseline for global health planning while underscoring the need for iterative updates and methodological refinement.

#### 4.4.1 Data quality and representativeness

While the GBD database offers extensive global coverage, the quality and representativeness of the data differ across regions. In low-resource settings, such as sub-Saharan Africa, South Asia, and certain areas of the Middle East, inadequate healthcare infrastructure, low diagnostic rates, and limited access to laparoscopic procedures may lead to an underestimation of the prevalence of endometriosis (45).

Li et al. highlighted that the data quality score for low-income countries in the GBD model is below 50%, suggesting that the actual prevalence rates may be 2–3 times higher than the reported estimates. These findings underscore that data collection and quality control remain major challenges in global endometriosis burden assessments (45).

Future research should focus on enhancing regional data collection and validation, integrating hospital-based case registries, community screening programs, and real-world clinical data to improve data accuracy in underserved regions. Additionally, machine learning-based data correction techniques could be employed to reduce underreporting and misclassification risks.

#### 4.4.2 Limitations of predictive models

This study utilized ARIMA and ES models to forecast the future burden of endometriosis. While these methods are effective for short-term trend predictions, they encounter several challenges when applied to long-term forecasting. ARIMA models, for instance, assume stationarity in time-series data; however, the epidemiological characteristics of endometriosis are subject to dynamic evolution due to advancements in diagnostics, changes in treatment approaches, and shifting disease trends. Such dynamic variations may result in nonlinear trends that traditional time-series models cannot adequately capture.

ES models heavily rely on historical trends and fail to account for external factors such as technological advancements, healthcare policies, and environmental influences, which can significantly impact the future burden of endometriosis.

To improve long-term prediction stability, future research should explore advanced modeling approaches, including: Long Short-Term Memory (LSTM) networks and Transformer-based models to capture complex temporal dependencies and predict nonlinear trends. Bayesian models can incorporate external predictors (e.g., healthcare policies, medical advancements) to refine disease burden estimates. Algorithms such as XGBoost can be applied to identify hidden nonlinear patterns in endometriosis burden projections.XGBoost. Future models should integrate environmental factors, medical advancements, and policy changes to enhance predictive accuracy and robustness.

## 5 Conclusion

This study systematically evaluates the global burden of endometriosis from 1990 to 2021, utilizing data from the Global Burden of Disease (GBD) 2021 database. Advanced statistical models were employed to project future trends through 2050.

Our findings underscore the critical importance of early diagnostic advancements, multidisciplinary management strategies, and the influence of environmental and socioeconomic factors on endometriosis epidemiology. Despite a decline in the overall incidence rate, endometriosis -related mortality and long-term disease burden continue to rise, presenting significant challenges for clinical management and public health systems.

## Data Availability

Publicly available datasets were analyzed in this study. This data can be found here: https://ghdx.healthdata.org/gbd-2021.
