# Supplementary figures and images for: Global burden of endometriosis from 1990 to 2021 and projections to 2050: a comprehensive analysis based on the global burden of disease study 2021
Source: Front Glob Womens Health. 2025 Oct 21;6:1613468. doi: 10.3389/fgwh.2025.1613468 (PMC12583065; doi:10.3389/fgwh.2025.1613468)

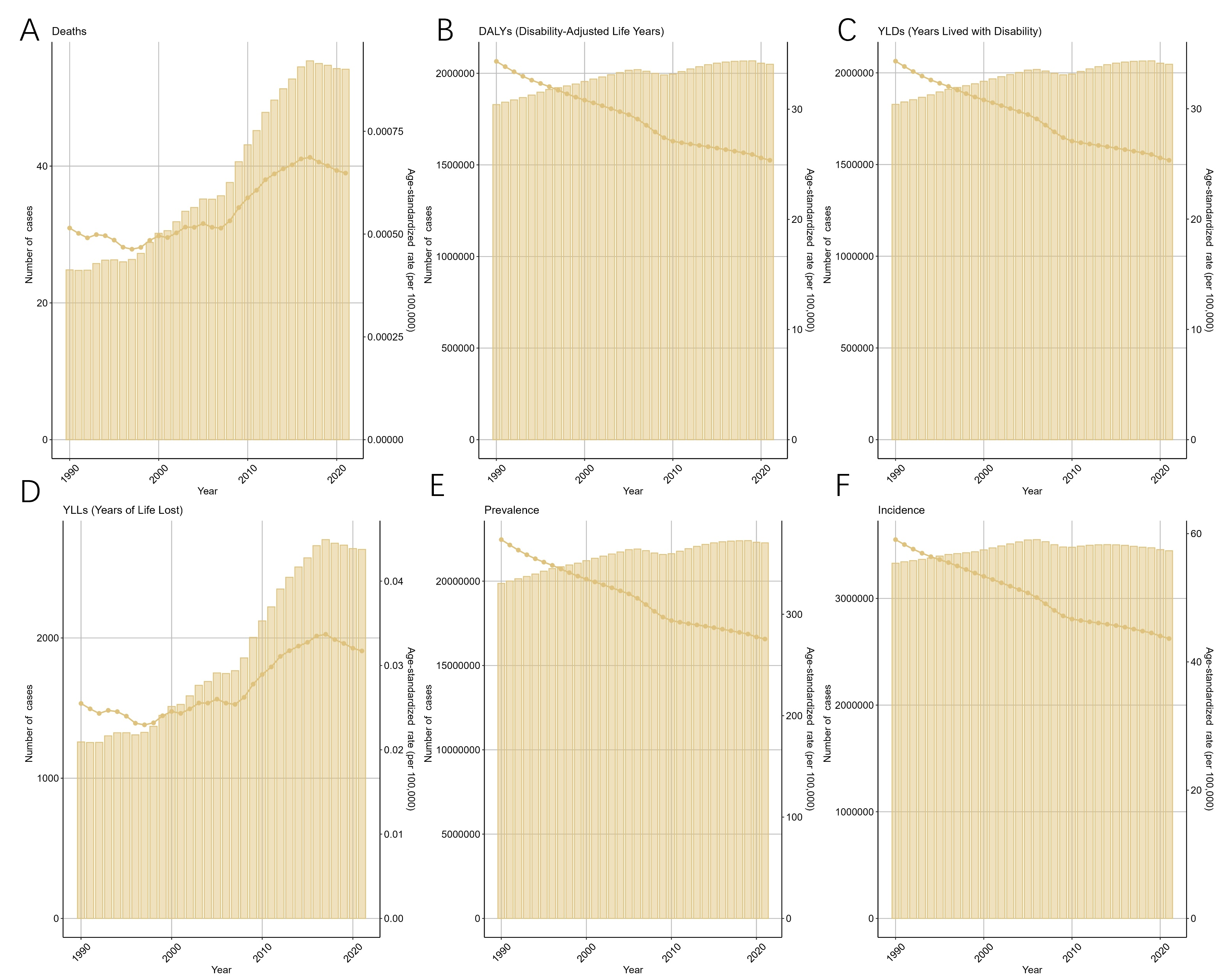

Supplement: Supplementary file 1 [file Image1.tif]

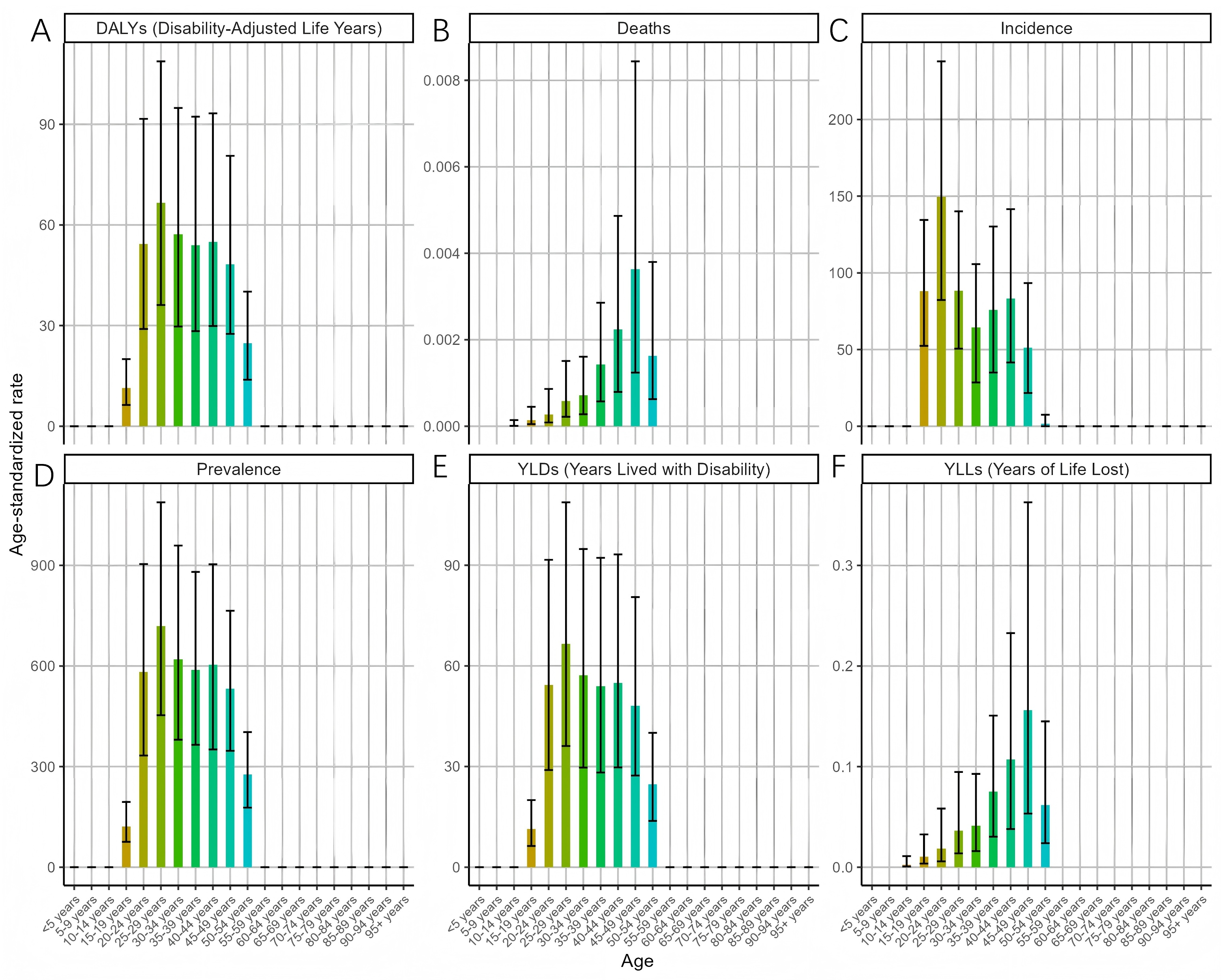

Supplement: Supplementary file 2 [file Image2.tif]

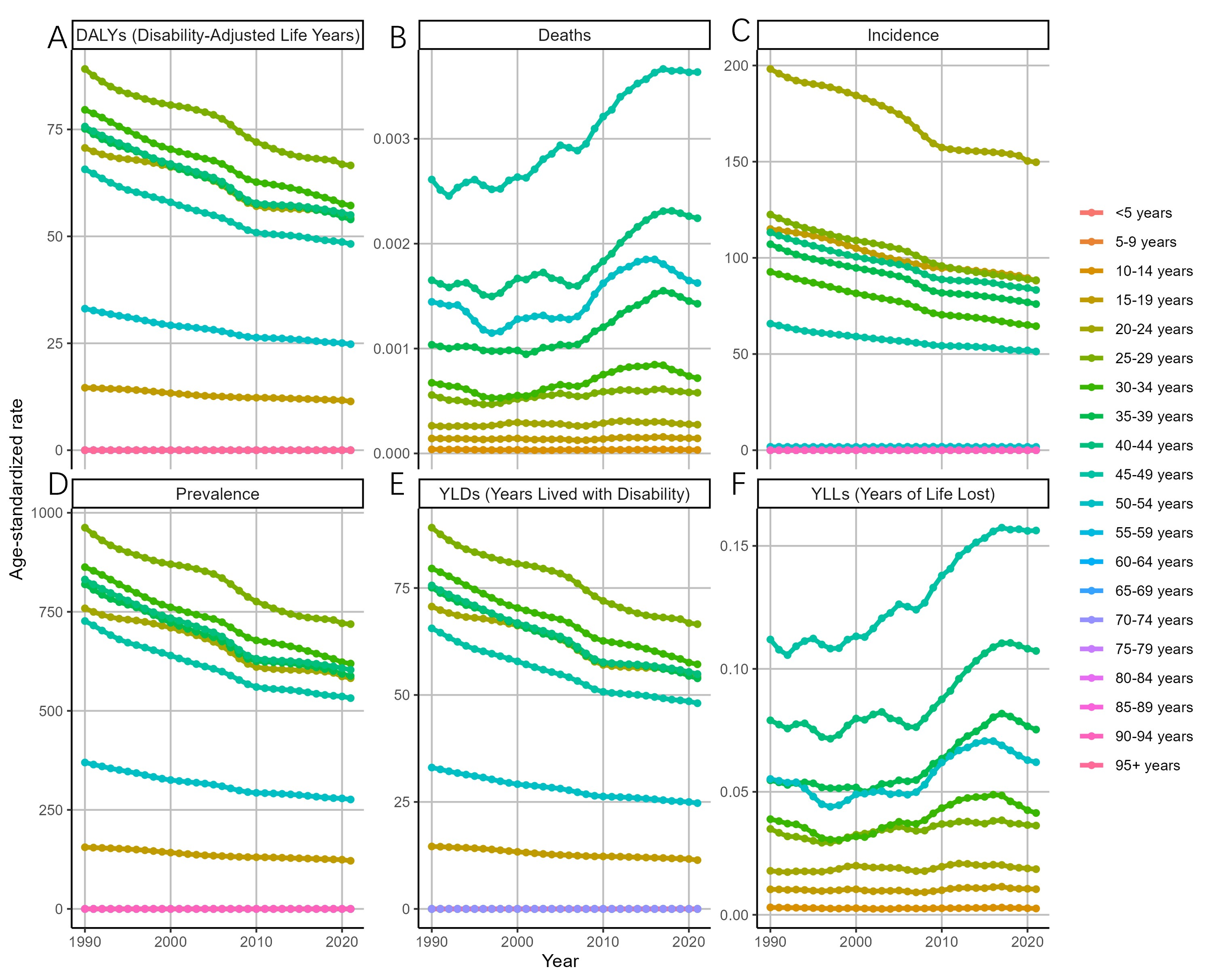

Supplement: Supplementary file 3 [file Image3.tif]

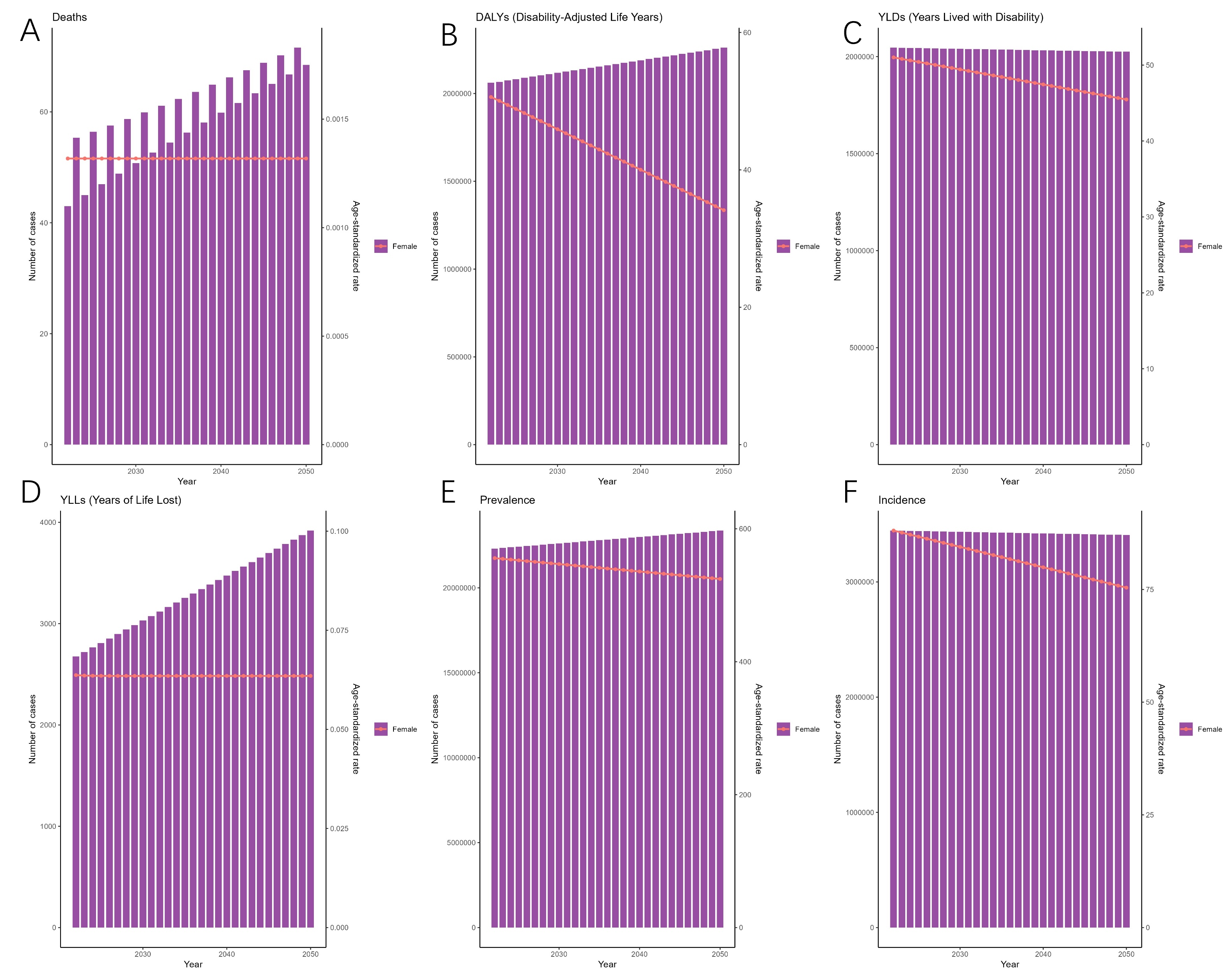

Supplement: Supplementary file 4 [file Image4.tif]

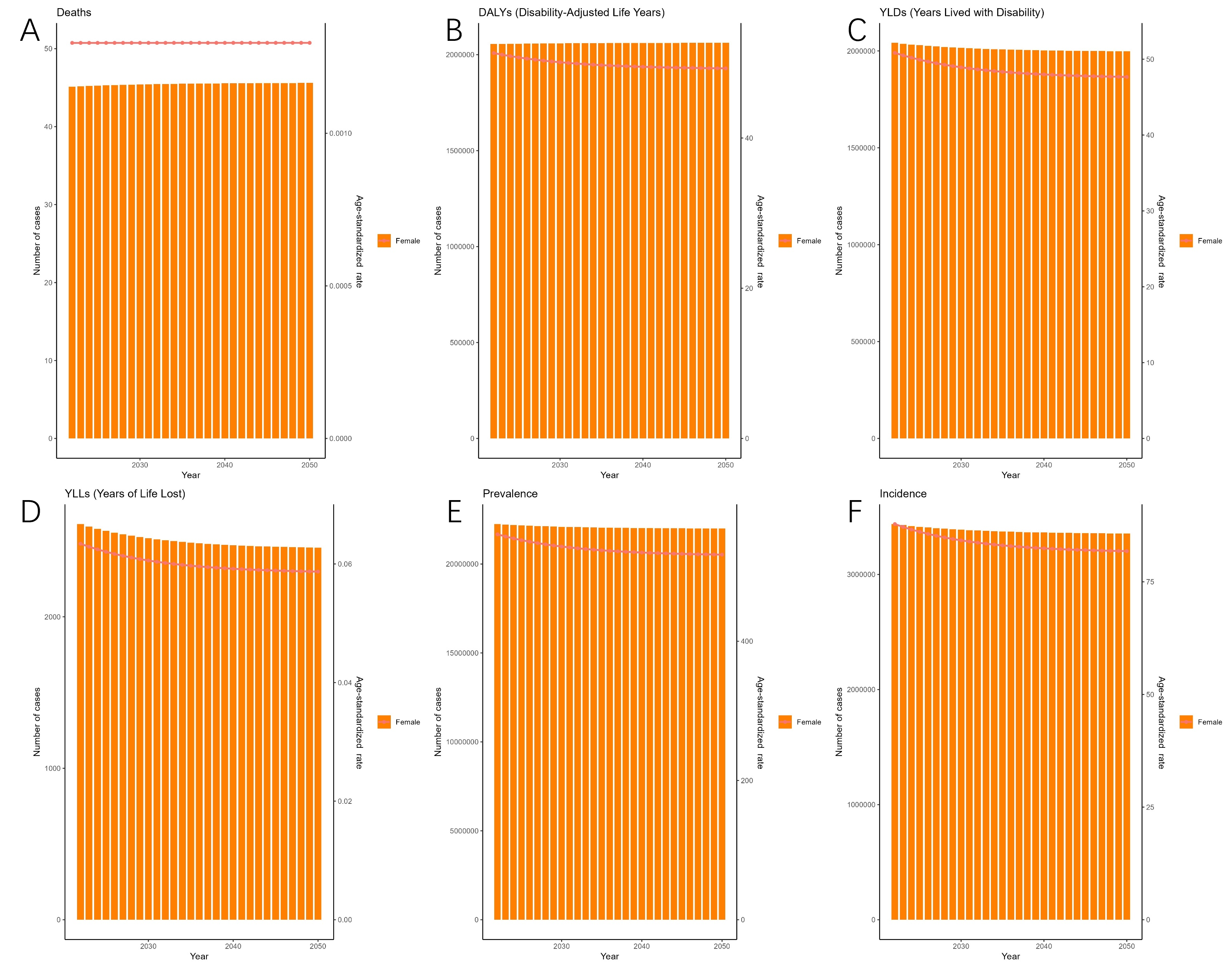

Supplement: Supplementary file 5 [file Image5.tif]
